# Supplementary material for: Removal of lycopene substrate inhibition enables high carotenoid productivity in Yarrowia lipolytica
Source: Nat Commun. 2022 Jan 31;13:572. doi: 10.1038/s41467-022-28277-w (PMC8803881; doi:10.1038/s41467-022-28277-w)
Supplement: Supplementary file 2 — Supplementary Information [file 41467_2022_28277_MOESM2_ESM.pdf]

Removal of lycopene substrate inhibition enables high carotenoid productivity in *Yarrowia lipolytica*

Ma *et al*.

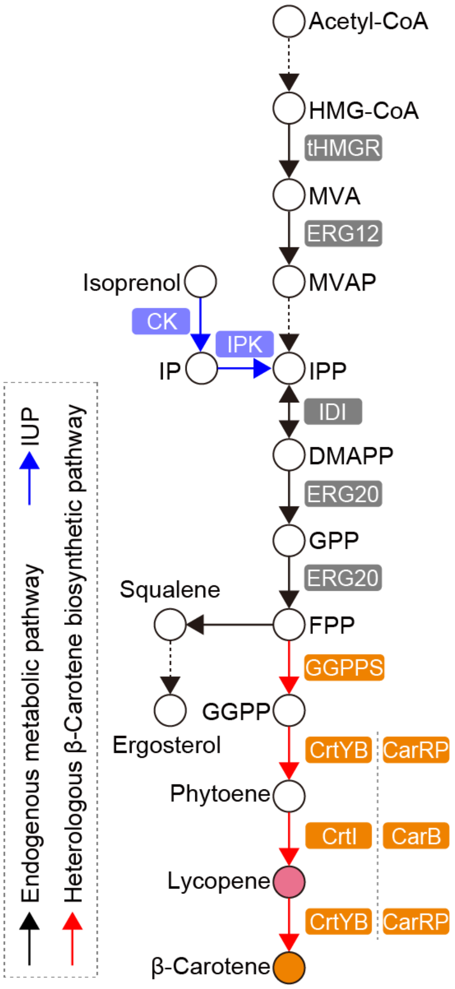

Supplementary Fig. 1. Scheme of the metabolic pathway leading to the production of β-carotene in *Y. lipolytica*. The engineered β-carotene biosynthetic pathway involved genes from the mevalonate pathway that were directly upregulated (gray), the isopentenol utilization pathway (IUP, blue), and β-carotene synthesis (orange). HMG-CoA, hydroxymethylglutaryl-CoA; MVA, mevalonate; MVAP, mevalonate-5-phosphate; IP, isopentenyl monophosphate; IPP, isopentenyl diphosphate; DMAPP, dimethylallyl diphosphate; GPP, geranyl pyrophosphate; FPP, farnesyl pyrophosphate; GGPP, geranylgeranyl pyrophosphate. tHMGR, truncated HMG-CoA reductase; ERG12, mevalonate kinase; IDI, isopentenyl diphosphate isomerase; ERG20, geranyl/farnesyl diphosphate synthase; GGPPS, GGPP synthase. CrtYB or CarRP, bi-functional phytoene synthase/lycopene β-cyclase; CrtI or CarB, phytoene dehydrogenase. CK, choline kinase; IPK, isopentenyl phosphate kinase.

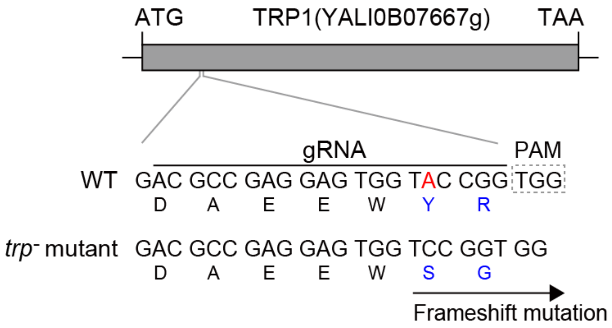

Supplementary Fig. 2. Disruption of *TRP1* in *Y. lipolytica* po1f strain via Crispr-Cas9. A single adenine deletion (red) in position -110 caused a frameshift mutation (blue) which abolished TRP1 activity.

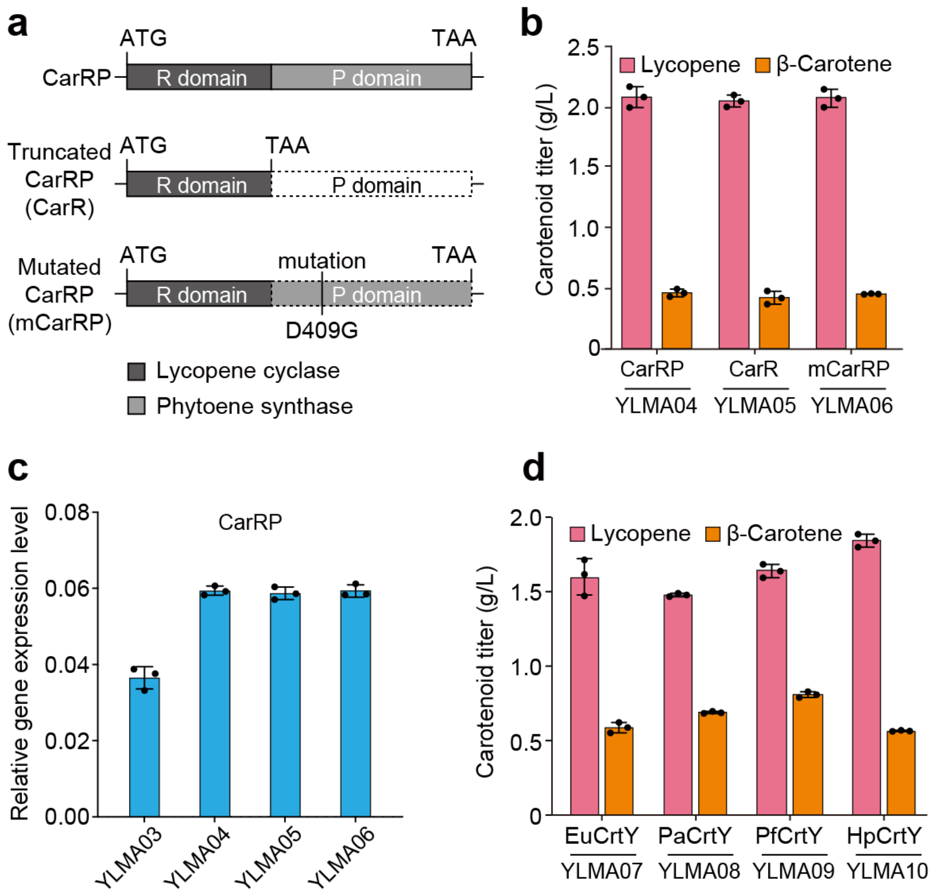

Supplementary Fig. 3. Effects of engineering the lycopene β-cyclase step on β-carotene production. a CarRP is a bi-functional enzyme with the R domain and P domain conferring lycopene cyclase and phytoene synthase activities, respectively. Two methods were used to isolate cyclase activity: truncation of the *CarRP* gene after the R domain and a loss of function mutation within the P domain (D409G). **b** Increasing the copy number of lycopene beta-cyclase did not improve β-carotene synthesis. **c** Relative gene expression level related to increased CarRP copy number. **d** Expressing lycopene β-cyclases from various other organisms led to improvements in β-carotene titers. However, the gains were minor and lycopene accumulation was still observed. The average and standard deviation (s.d.) of three biologically independent experiments are shown. Source data are provided as a Source Data file.

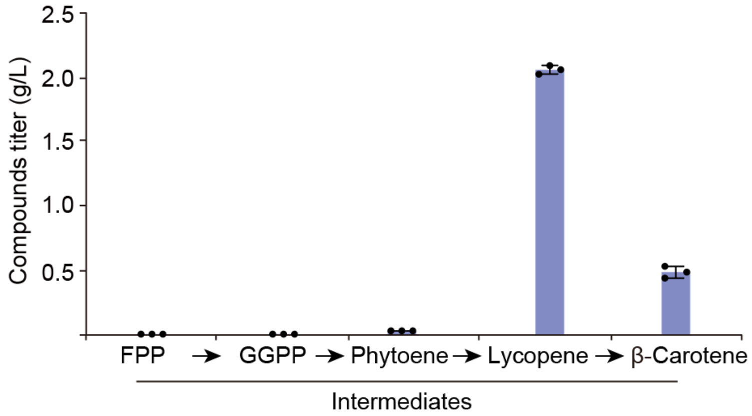

Supplementary Fig. 4. Intermediate levels involved in β-carotene synthetic pathway. The intracellular concentrations of biosynthetic intermediates from FPP to β-carotene were measured, and lycopene was the only aggregating precursor. Data shown are the average and s.d. of three biologically independent experiments. Source data are provided as a Source Data file.

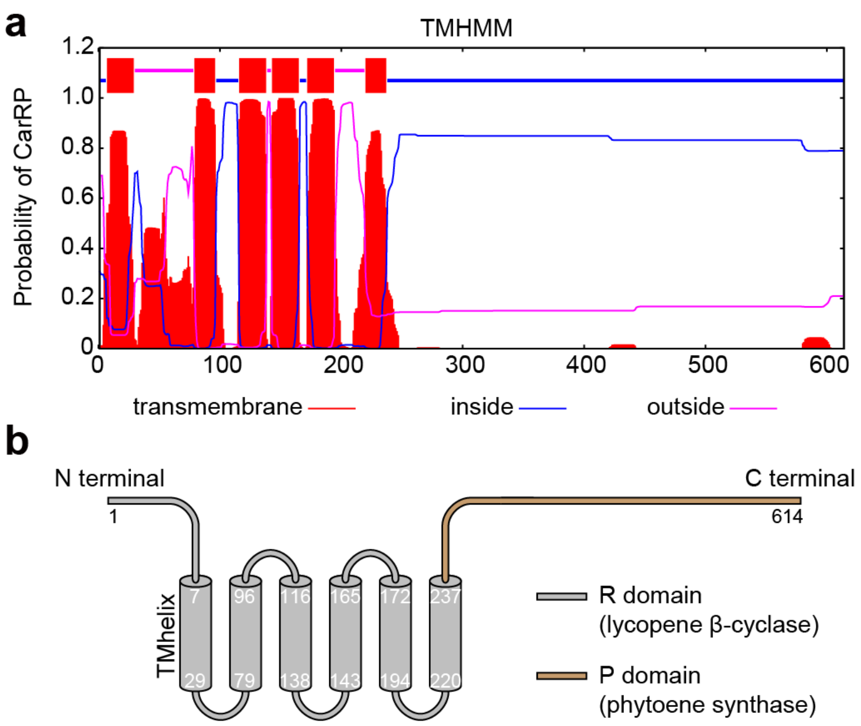

Supplementary Fig. 5. Structural predictions of the lycopene beta-cyclase in CarRP. a Presence of the 6 transmembrane domains (red) in CarRP was calculated using the TMHMM server v. 2.0. **b** A schematic of CarRP protein showing that the 6 transmembrane helices are within the R domain (β-cyclase).

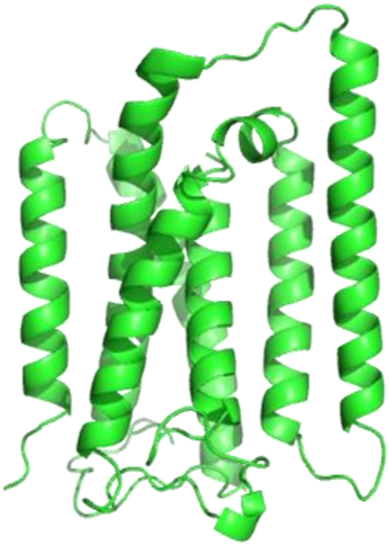

Supplementary Fig. 6. Computational model of R domain (lycopene cyclase) of CarRP. The model having TrRosetta confidence score is 0.79, suggesting a high certainty in structural model.

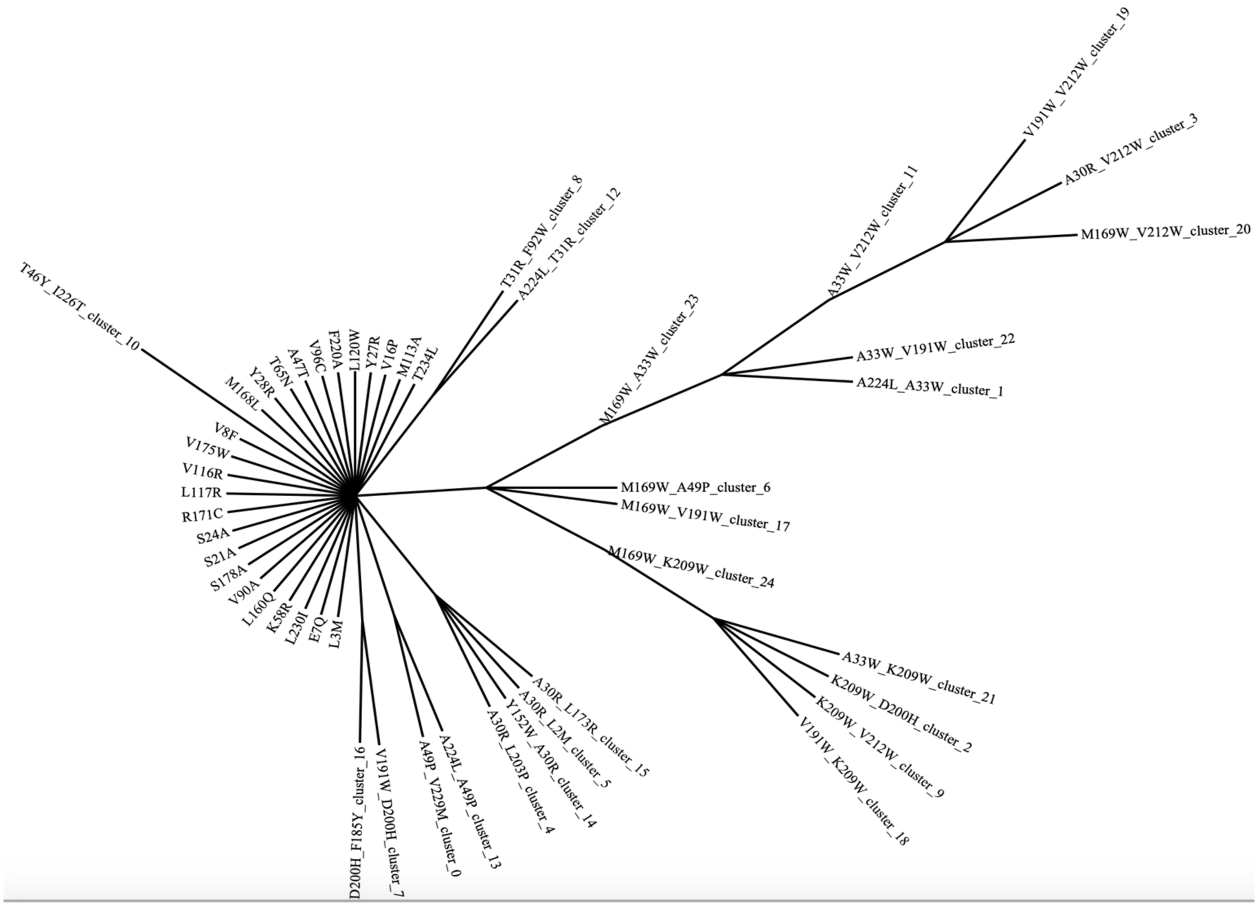

Supplementary Fig. 7. Clustering of variants to illustrate distance between sequences. The variants were clustered using PhyML[1] to ensure spread of variants being tested.

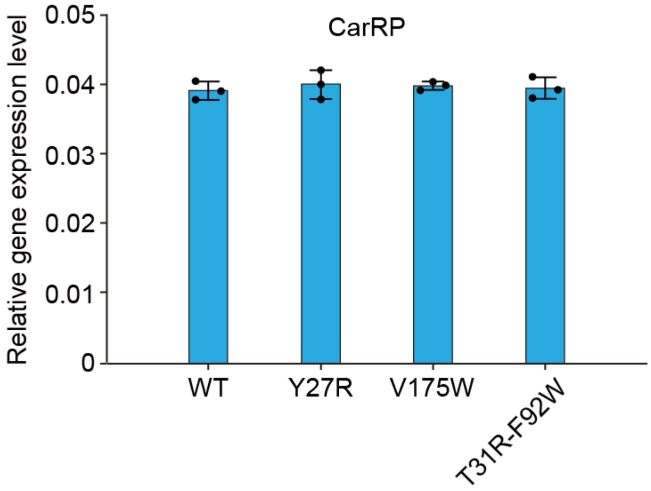

Supplementary Fig. 8. Relative gene expression level related to the CarRP variants. Relative expression levels of gene CarRP in engineered strains harboring mutated CarRP as well as wide type CarRP (WT) were quantified by RT-PCR. ACT1 was used as an internal control gene for normalization. Data shown are the average and s.d. of three biologically independent experiments. Source data are provided as a Source Data file.

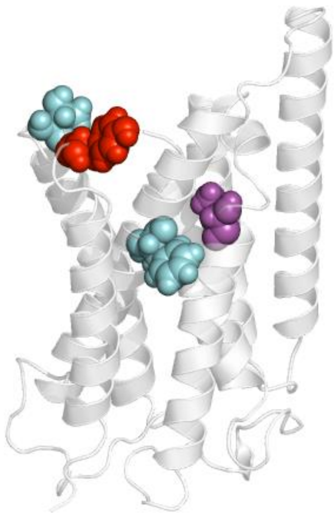

Supplementary Fig. 9. Spatial mapping of the substitutions removing substrate inhibition. The positions of the success variants are mapped onto the computational model of the lycopene cyclase with Y27R (red), V175W (magenta), and T31R-F92W (cyan) shown in spheres. All substitutions seemed to be located in same spatial area of the enzyme.

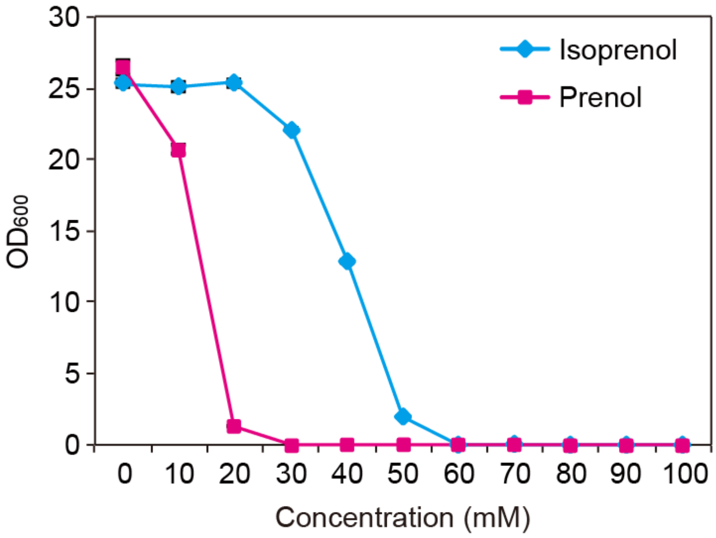

Supplementary Fig. 10. The effect of isoprenol or prenol on cell growth. Isopentenol isomers isoprenol (blue) or prenol (red) were fed to a po1f strain in YPD media at varying concentrations. OD_{600} was measured after 24h of cultivation. Based on these results, 30 mM isoprenol or 10 mM prenol was found to be the suitable concentration for all subsequent experiments. Data shown are the average and s.d. of three biologically independent experiments. Source data are provided as a Source Data file.

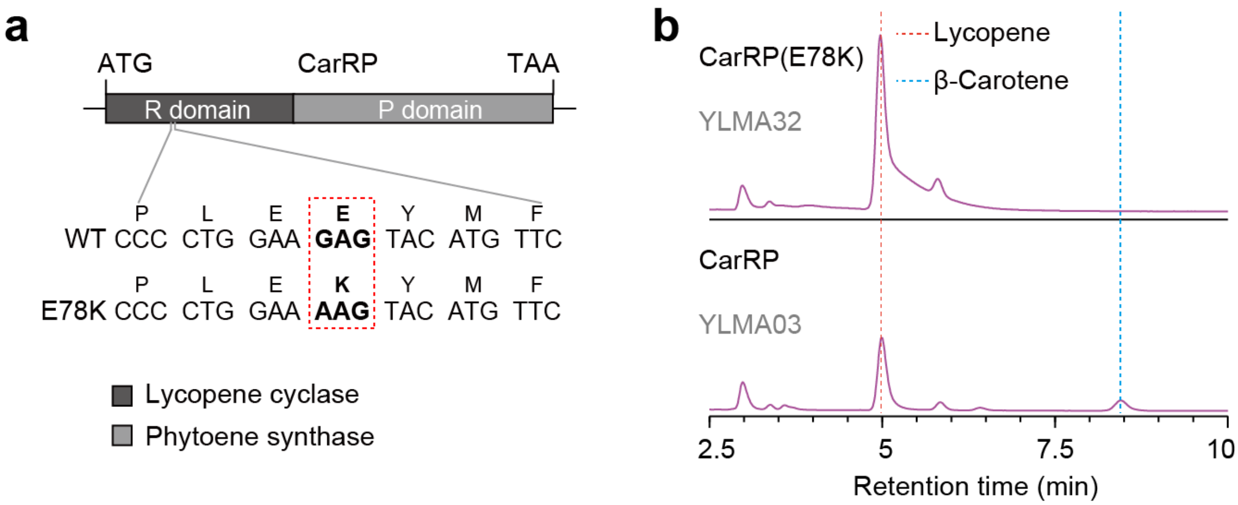

Supplementary Fig. 11. Disruption of lycopene cyclase activity in CarRP. **a** The E78K mutation in the R domain of CarRP as indicated by red dotted rectangle leads to a loss of function of cyclase activity. **b** The HPLC chromatographs showed that mutated $CarRP^{E78K}$ completely abolished β-carotene formation.

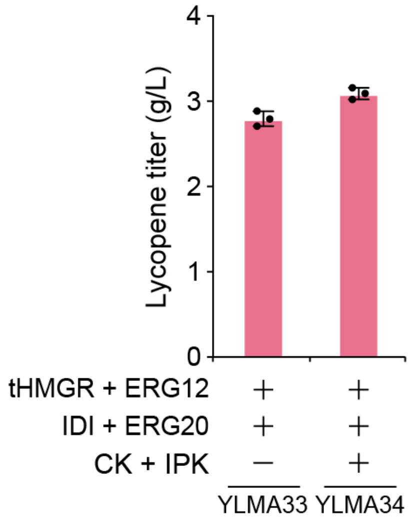

Supplementary Fig. 12. Engineering MVA pathway and IUP further promotes lycopene biosynthesis. Overexpressing the native genes in MVA pathway improved lycopene synthesis. Furthermore, additional introduction of IUP in the lycopene-producing strains further improved titers. Data shown are the average and s.d. of three biologically independent experiments. Source data are provided as a Source Data file.

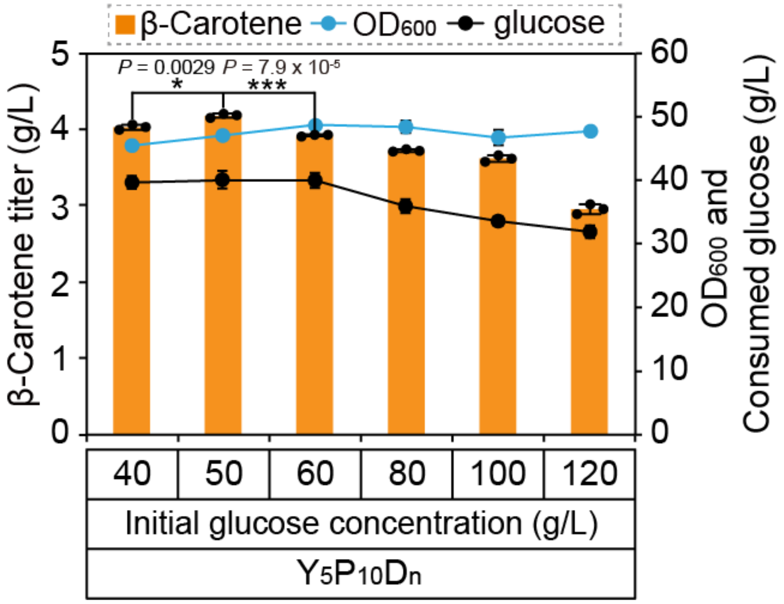

Supplementary Fig. 13. Determination of the optimal initial glucose concentration for β-carotene production. Cells were cultured in $Y_5P_{10}D_n$ media where n represents initial glucose concentration. After 3 days of fermentation, the amount of glucose consumed, OD_{600}, and β-carotene titers were measured. The optimal initial glucose concentration was found to be 50 g/L. Beyond that, the performance of the strain was adversely affected and glucose consumption rates decreased as well, presumably due to osmotic stress. The average and s.d. of three biologically independent experiments are shown. Statistical differences were analyzed using two-sided Student's t-test, and $P < 0.05$ was considered to be statistically significant. *$P < 0.05$, ***$P < 0.001$. Source data are provided as a Source Data file.

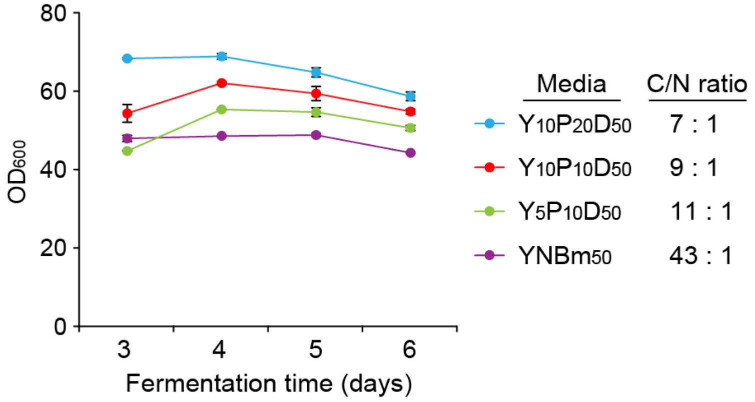

Supplementary Fig. 14. Comparison of cell growth in media with varying C/N ratios. In these experiments, the YLMA15 strain was used and biomass was found to decrease with increasing C/N ratio. Data shown are the average and s.d. of three biologically independent experiments. Source data are provided as a Source Data file.

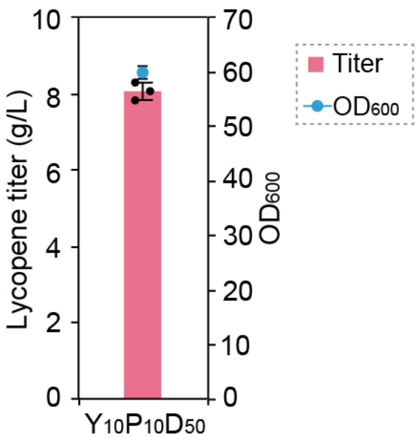

Supplementary Fig. 15. Using the optimized $Y_{10}P_{10}D_{50}$ media, lycopene titers were further enhanced. Data shown are the average and s.d. of three biologically independent experiments. Source data are provided as a Source Data file.

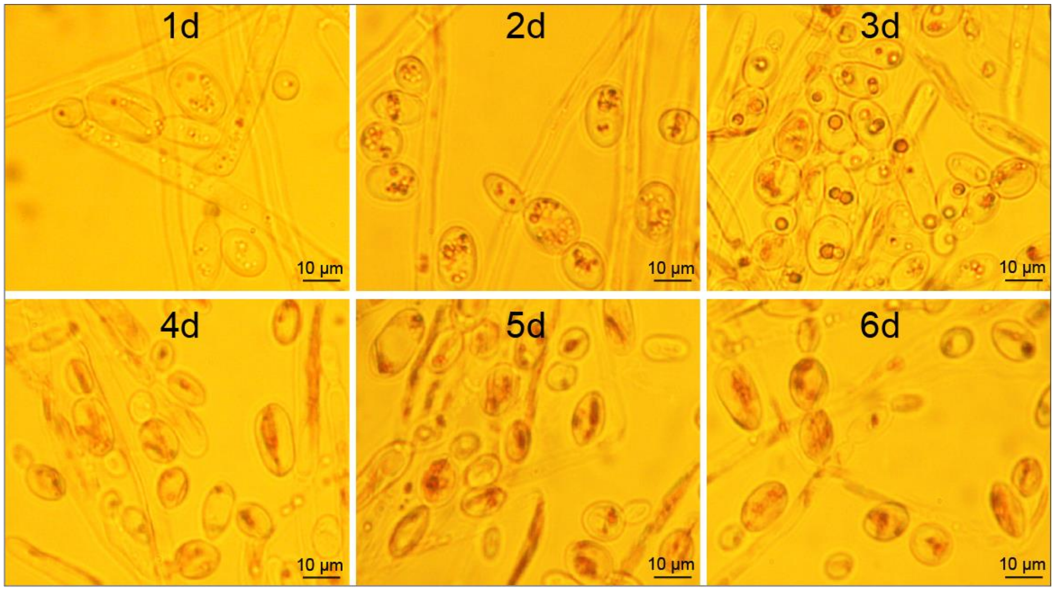

Supplementary Fig. 16. The micro-morphology of YLMA15 cells throughout fermentation. Cells collected at different time points throughout cultivation were visualized under a microscope and the observations were consistent with the fermentation profile. In the present of glucose in media during the initial 3 days, lipid droplets within cells progressively agglomerated into lipid bodies that sequestered the produced β-carotene. However, due to TAG breakdown, the lipid bodies were no longer visible during the later stages of glucose-depletion, which in turn caused the accumulated β-carotene to be more dispersed throughout the cell. Three independent experiments were repeated with similar results.

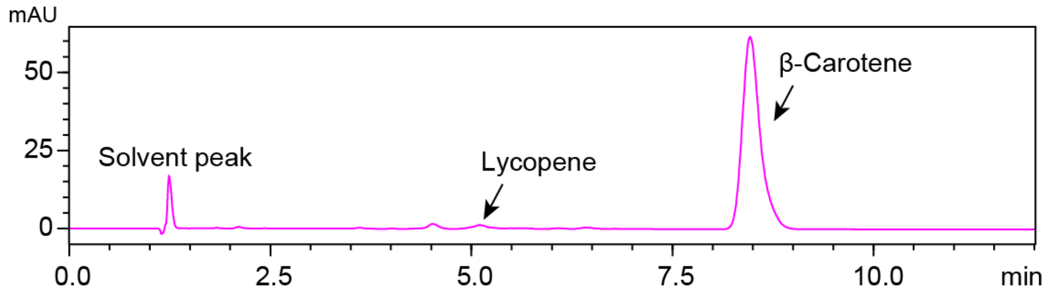

Supplementary Fig. 17. The HPLC chromatograph of carotenoids obtained from YLMA15 after fed-batch fermentation. The selectivity of β-carotene was calculated based on the relative contents of lycopene and β-carotene.

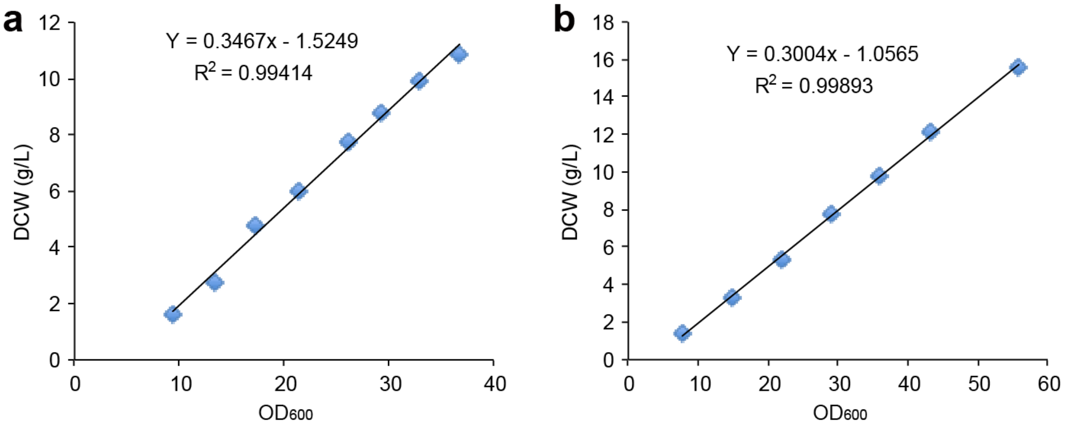

Supplementary Fig. 18. Correlation between dry cell weight (DCW) and OD_{600} in β-carotene-producing cells (a), and lycopene-producing cells (b). DCW was calculated based on the measured OD_{600} and applying the conversion factor. Source data are provided as a Source Data file.

Supplementary Table 1. List of heterologous enzymes used in this study.

Enzyme	Name used in this study	Accession No.	Organism
Phytoene dehydrogenase	CrtI	AY177424.1	*Xanthophyllomyces dendrorhous*
	CarB	AJ238028.1	*Mucor circinelloides*
Phytoene synthase /lycopene cyclase	CrtYB	AY177204.1	*Xanthophyllomyces dendrorhous*
	CarRP	AJ250827.1	*Mucor circinelloides*
GGPP synthase	GGPPpa	AAA21260.1	*Pantoea agglomerans*
	GGPPtc	AF081514.1	*Taxus canadensis*
	GGPPsa	D28748.1	*Sulfolobus acidocaldarius*
	GGPPxd	DQ016502.1	*Xanthophyllomyces dendrorhous*
	GGPPyl	XM_502923.1	*Yarrowia lipolytica*
lycopene beta-cyclase	EuCrtY	D90087.2	*Erwinia uredovora*
	PaCrtY	JX871358.1	*Pantoea agglomerans*
	PfCrtY	CP002727.1	*Pseudomonas fulva*
	HpCrtY	AY182008.1	*Haematococcus pluvialis*

AY177424.1 (https://www.ncbi.nlm.nih.gov/nuccore/AY177424.1/);

AJ238028.1 (https://www.ncbi.nlm.nih.gov/nuccore/AJ238028.1);

AY177204.1 (https://www.ncbi.nlm.nih.gov/nuccore/AY177204.1);

AJ250827.1 (https://www.ncbi.nlm.nih.gov/nuccore/AJ250827.1);

AAA21260.1 (https://www.ncbi.nlm.nih.gov/protein/AAA21260.1);

AF081514.1 (https://www.ncbi.nlm.nih.gov/nuccore/AF081514.1);

D28748.1 (https://www.ncbi.nlm.nih.gov/nuccore/D28748.1);

DQ016502.1 (https://www.ncbi.nlm.nih.gov/nuccore/DQ016502.1);

XM_502923.1 (https://www.ncbi.nlm.nih.gov/nuccore/XM_502923.1);

D90087.2 (https://www.ncbi.nlm.nih.gov/nuccore/D90087.2);

JX871358.1 (https://www.ncbi.nlm.nih.gov/nuccore/JX871358.1);

CP002727.1 (https://www.ncbi.nlm.nih.gov/nuccore/CP002727.1);

AY182008.1 (https://www.ncbi.nlm.nih.gov/nuccore/AY182008.1).

Supplementary Table 2. Compositions of modified YPD and YNB media used in this study.

	**Yeast extract (g/L)**	**Peptone (g/L)**	**Glucose (g/L)**	**C/N ratio**
$Y_{10}P_{20}D_{50}$	10	20	50	7:1
$Y_{10}P_{10}D_{50}$	10	10	50	9:1
$Y_{5}P_{10}D_{50}$	5	10	50	11:1
	**Yeast extract (g/L)**	**YNB (g/L)**	**Glucose (g/L)**	**C/N ratio**
$YNBm_{50}$	5	6.9	50	43:1

Supplementary Table 3. Summary of carotenoid production in microorganisms.

Carotenoids	Host	Fermentation scale	Titer (g/L)	Content (mg/g DCW)	Productivity (g/L/h)[a]	References
β-carotene	*Y. lipolytica*	3 L fermenter	39.5	494	0.165	In this study
		5 L fermenter	6.5	90	0.045	[2]
		2 L fermenter	4	50	0.014	[3]
	S. cerevisiae	5 L fermenter	1.2	21	0.01	[4]
	E. coli	5 L fermenter	3.2	-	0.04	[5]
		7 L fermenter	2.1	60	0.021	[6]
		5 L fermenter	2	-	0.015	[7]
Lycopene	*Y. lipolytica*	3 L fermenter	17.6	313	0.073	In this study
		3 L fermenter	4.2	-	0.016	[8]
	S. cerevisiae	7 L fermenter	3.28	-	0.02	[9]
		7 L fermenter	2.37	73.3	0.018	[10]
		7 L fermenter	2.3	-	0.015	[11]
		5 L fermenter	1.65	55.6	0.014	[12]
		5 L fermenter	1.61	24.4	0.013	[13]
	E. coli	7 L fermenter	3.52	50.6	0.029	[14]

[a]Productivity is calculated as the total carotenoid produced divided by total fermentation time.

Supplementary Table 4. Strains and constructs used in this study.

Strain name	Description of strains and plasmids	Source
po1f	*MatA*, *leu2-270*, *ura3-302*, *xpr2-322*, *axp-2*	Lab stock
po1f-T	*MatA*, *leu2-270*, *ura3-302*, *xpr2-322*, *axp-2*, *trp1*	This study
YLMA01	po1f-T, *URA3*:: T_{Lip1}-*CrtI*-P_{GPD}+P_{TEF}-*CrtYB*-T_{XPR2}	This study
YLMA02	po1f-T, *URA3*:: T_{Lip1}-*CarB*-P_{GPD}+P_{TEF}-*CarRP*-T_{XPR2}	This study
YLMA03	YLMA02, *LEU2*:: P_{TEF}-*GGPPxd*-T_{XPR2}	This study
YLMA04	YLMA03, *TRP1*:: P_{TEF}-*CarRP*-T_{XPR2}	This study
YLMA05	YLMA03, *TRP1*:: P_{TEF}-t*CarRP*-T_{XPR2}	This study
YLMA06	YLMA03, *TRP1*:: P_{TEF}-m*CarRP*-T_{XPR2}	This study
YLMA07	YLMA03, *TRP1*:: P_{TEF}-*EuCrtY*-T_{XPR2}	This study
YLMA08	YLMA03, *TRP1*:: P_{TEF}-*PaCrtY*-T_{XPR2}	This study
YLMA09	YLMA03, *TRP1*:: P_{TEF}-*PfCrtY*-T_{XPR2}	This study
YLMA10	YLMA03, *TRP1*:: P_{TEF}-*HpCrtY*-T_{XPR2}	This study
YLMA11	po1f-T, *LEU2*:: P_{TEF}-*GGPPxd*-T_{XPR2}+T_{Lip1}-*CarB*-P_{GPD}+P_{TEF}-$CarRP^{Y27R}$-T_{XPR2}	This study
YLMA12	po1f-T, *LEU2*:: P_{TEF}-*GGPPxd*-T_{XPR2}+T_{Lip1}-*CarB*-P_{GPD}+P_{TEF}-$CarRP^{V175W}$-T_{XPR2}	This study
YLMA13	po1f-T, *LEU2*:: P_{TEF}-*GGPPxd*-T_{XPR2}+T_{Lip1}-*CarB*-P_{GPD}+P_{TEF}-$CarRP^{T31R\text{-}F92W}$-T_{XPR2}	This study
YLMA14	YLMA11, *URA3*:: T_{Lip1}-*ERG12*-P_{EXP1}+P_{TEFin}-*tHMGR*-T_{XPR2}+ T_{Lip1}-*ERG20*-P_{EXP1}+P_{TEFin}-*IDI*-T_{XPR2}	This study
YLMA15	YLMA14, *TRP1*:: T_{Lip1}-*CK*-P_{EXP1}+P_{TEFin}-*IPK*-T_{XPR2}	This study
YLMA16	po1f-T, *TRP1*:: T_{Lip1}-*CK*-P_{EXP1}+P_{TEFin}-*IPK*-T_{XPR2}	This study
YLMA17	po1f, *LEU2*:: P_{TEF}-*GGPPtc*-T_{XPR2}	This study
YLMA18	po1f, *LEU2*:: P_{TEF}-*GGPPpa*-T_{XPR2}	This study
YLMA19	po1f, *LEU2*:: P_{TEF}-*GGPPyl*-T_{XPR2}	This study
YLMA20	po1f, *LEU2*:: P_{TEF}-*GGPPsa*-T_{XPR2}	This study
YLMA21	po1f, *LEU2*:: P_{TEF}-*GGPPxd*-T_{XPR2}	This study

YLMA22	YLMA02, *LEU2*:: P_{TEF}-*GGPPtc*-T_{XPR2}	This study
YLMA23	YLMA02, *LEU2*:: P_{TEF}-*GGPPpa*-T_{XPR2}	This study
YLMA24	YLMA02, *LEU2*:: P_{TEF}-*GGPPyl*-T_{XPR2}	This study
YLMA25	YLMA02, *LEU2*:: P_{TEF}-*GGPPsa*-T_{XPR2}	This study
YLMA26	po1f-T, *LEU2*:: P_{TEF}-*GGPPsa*-T_{XPR2}+T_{Lip1}-*CarB*-P_{GPD}+P_{TEF}-*CarRP*-T_{XPR2}	This study
YLMA27	YLMA26, *TRP1*:: P_{TEFin}-*GGPPsa*-T_{XPR2}+T_{Lip1}-*CarB*-P_{EXP1}+P_{TEFin}-*CarRP*-T_{XPR2}	This study
YLMA28	YLMA27, *URA3*:: P_{TEFin}-*GGPPsa*-T_{XPR2}+T_{Lip1}-*CarB*-P_{EXP1}+P_{TEFin}-*CarRP*-T_{XPR2}	This study
YLMA29	YLMA28, $ura^{-}/leu^{-}/trp^{-}$	This study
YLMA30	YLMA29, *URA3*:: T_{Lip1}-*ERG12*-P_{EXP1}+P_{TEFin}-*tHMGR*-T_{XPR2}+ T_{Lip1}-*ERG20*-P_{EXP1}+P_{TEFin}-*IDI*-T_{XPR2}	This study
YLMA31	YLMA30, *TRP1*:: T_{Lip1}-*CK*-P_{EXP1}+P_{TEFin}-*IPK*-T_{XPR2}	This study
YLMA32	po1f-T, *LEU2*:: P_{TEF}-*GGPPxd*-T_{XPR2}+T_{Lip1}-*CarB*-P_{GPD}+P_{TEF}-$CarRP^{E78K}$-T_{XPR2}	This study
YLMA33	YLMA32, *URA3*:: T_{Lip1}-*ERG12*-P_{EXP1}+P_{TEFin}-*tHMGR*-T_{XPR2}+ T_{Lip1}-*ERG20*-P_{EXP1}+P_{TEFin}-*IDI*-T_{XPR2}	This study
YLMA34	YLMA33, *TRP1*:: T_{Lip1}-*CK*-P_{EXP1}+P_{TEFin}-*IPK*-T_{XPR2}	This study

Supplementary Table 5. Primers used in this study.

Crispr-trp-F	GGGTCGGCGCAGGTTGACGTACGCCGAGGAGTGGTACCGGGTTTTAGAGCTAGAAATAGC
Crispr-trp-R	GCTATTTCTAGCTCTAAAACCCGGTACCACTCCTCGGCGTACGTCAACCTGCGCCGACCC
CrtI-F	ATGAACCAGATGCATAGCACTTAGAAGGCCAGGATGCCCA
CrtI-R	CATCAACAGTATCTACACGCATGGGCAAGGAACAGGACCA
CrtYB-F	CATTCAAAGGATCCCCTAGGATGACCGCTCTGGCCTACTA
CrtYB-R	GCAAGACCGGCAACGTGGGGTTACTGGCCCTCCCAGCCAG
CarB-F	ATGAACCAGATGCATAGCACTTAGATCACGTTAGAGTTGT
CarB-R	CATCAACAGTATCTACACGCATGTCCAAGAAGCACATCGT
CarRP-F	CATTCAAAGGATCCCCTAGGATGCTGCTGACCTACATGGA
CarRP-R	GCAAGACCGGCAACGTGGGGTTAGATGGTGTTCAGGTTTC
GGPPxd-F	CATTCAAAGGATCCCCTAGGATGGACTACGCCAACATCCT
GGPPxd-R	GCAAGACCGGCAACGTGGGGTTACAGGGGAATGTCGGCCA
tCarRP-F	CATTCAAAGGATCCCCTAGGATGCTGCTGACCTACATGGA
tCarRP-R	GCAAGACCGGCAACGTGGGGTTACAGCTCTTGTCGCACGTCG
mCarRP-1-F	CATTCAAAGGATCCCCTAGGATGCTGCTGACCTACATGGA
mCarRP-1-R	TCGCTCGAGGCCCCACTTGTAGCCGTCCAG
mCarRP-2-F	ACAAGTGGGGCCTCGAGCGACGATCTATCC
mCarRP-2-R	GCAAGACCGGCAACGTGGGGTTAGATGGTGTTCAGGTTTC
EuCrtY-F	CATTCAAAGGATCCCCTAGGATGCAGCCCCACTACGACCT
EuCrtY-R	GCAAGACCGGCAACGTGGGGTTATCGGTGAGTGGTCATGA
PaCrtY-F	CATTCAAAGGATCCCCTAGGATGCCCCGATACGACCTGAT
PaCrtY-R	GCAAGACCGGCAACGTGGGGTTACTGCATGGCCTGCTGTC
PfCrtY-F	CATTCAAAGGATCCCCTAGGATGTCTTACGACCTGATCCT
PfCrtY-R	GCAAGACCGGCAACGTGGGGTTAAGATCGCATCTCGAAGT
HpCrtY-F	CATTCAAAGGATCCCCTAGGATGCTGTCTCCCCTGCAGCG
HpCrtY-R	GCAAGACCGGCAACGTGGGGTTACTTGATCATGGCTGGAG

GGPPtc-F	CATTCAAAGGATCCCCTAGGATGGCCTACACCGCCATGGC
GGPPtc-R	GCAAGACCGGCAACGTGGGGTTAGTTCTGTCGGAAGGCAA
GGPPpa-F	CATTCAAAGGATCCCCTAGGATGGTCTCTGGATCTAAGGC
GGPPpa-R	GCAAGACCGGCAACGTGGGGTCAGGCAATCTTCATGACAG
GGPPyl-F	CATTCAAAGGATCCCCTAGGATGGATTATAACAGCGCGGA
GGPPyl-R	GCAAGACCGGCAACGTGGGGTCACTGCGCATCCTCAAAGT
GGPPsa-F	CATTCAAAGGATCCCCTAGGATGTCCTACTTCGACAACTA
GGPPsa-R	GCAAGACCGGCAACGTGGGGTTACTTTCGTCGTCGAATGG
ERG12-F	ATGAACCAGATGCATAGCACCTAATGGGTCCAGGGACCGA
ERG12-R	ACACAAGACATATCTACAGCATGGACTACATCATTTCGGC
tHMGR-F	TTTTGCAGTACTAACCGCAGACCCAGTCTGTGAAGGTGGT
tHMGR-R	GCAAGACCGGCAACGTGGGGCTATGACCGTATGCAAATAT
ERG20-F	ATGAACCAGATGCATAGCACCTACTTCTGTCGCTTGTAAA
ERG20-R	ACACAAGACATATCTACAGCATGTCCAAGGCGAAATTCGA
IDI-F	TTTTGCAGTACTAACCGCAGACGACGTCTTACAGCGACAA
IDI-R	GCAAGACCGGCAACGTGGGGCTACTTGATCCACCGCCGAA
CK-F	ATGAACCAGATGCATAGCACCTACGCATTAATAAACCGGG
CK-R	ACACAAGACATATCTACAGCATGGCCCCTCAACCCGACCA
IPK-F	TTTTGCAGTACTAACCGCAGGAACTGAACATCTCTGAGTC
IPK-R	GCAAGACCGGCAACGTGGGGTTACTTGGAGAATCGGATGA
ACT-RT-F	GAGTCACCGGTATCGTTC
ACT-RT-R	GCGGAGTTGGTGAAAGAG
CarRP-RT-F	CTGGCCCTGCTGTGGTTCGG
CarRP-RT-R	CAGGCAGATGGGGCACCACG

Supplementary Table 6. Composition of Yeast extract (Bacto™) and Peptone (Bacto™).

Composition	Yeast extract	Peptone	Composition	Yeast extract	Peptone
**Total Nitrogen (%)**	**10.9**	**15.4**	Glutamine (% Free)	0.2	0.0
Amino Nitrogen (%)	6.0	3.5	Glycine (% Free)	1.0	0.7
AN/TN	0.55	0.20	Glycine (% Total)	3.0	15.9
Total Carbohydrate (mg/g)	163.3	6.29	Histidine (% Free)	0.4	0.2
Ash (%)	11.2	3.8	Histidine (% Total)	1.3	0.8
Loss on Drying (%)	3.1	2.7	Isoleucine (% Free)	1.8	0.6
NaCl (%)	0.1	1.7	Isoleucine (% Total)	3.0	2.1
pH (1% Solution)	6.7	7.1	Leucine (% Free)	3.0	1.6
Calcium (µg/g)	130	30	Leucine (% Total)	4.1	3.8
Iron (µg/g)	55.3	7.8	Lysine (% Free)	1.9	2.2
Magnesium (µg/g)	750	17	Lysine (% Total)	4.6	3.4
Potassium (µg/g)	31950	2487	Methionine (% Free)	0.6	0.3
Sodium (µg/g)	4900	18127	Methionine (% Total)	0.8	0.7
Chloride (%)	0.38	0.90	Phenylalanine (% Free)	2.0	1.4
Sulfate (%)	0.09	0.32	Phenylalanine (% Total)	2.6	2.8
Phosphate (%)	3.27	0.40	Proline (% Free)	0.8	0.3
Alanine (% Free)	4.4	1.2	Proline (% Total)	2.0	8.8
Alanine (% Total)	5.6	9.2	Serine (% Free)	1.3	0.4
Arginine (% Free)	1.4	2.8	Serine (% Total)	1.6	1.5
Arginine (% Total)	2.6	5.8	Threonine (% Free)	1.1	0.3
Asparagine (% Free)	1.0	0.3	Threonine (% Total)	1.6	1.1
Aspartic Acid (% Free)	1.6	0.3	Tryptophan (% Free)	0.5	0.3
Aspartic Acid (% Total)	5.3	5.0	Tyrosine (% Free)	0.8	0.5
Cystine (% Free)	0.2	0.0	Tyrosine (% Total)	1.2	0.6
Glutamic Acid (% Free)	6.6	0.7	Valine (% Free)	2.2	0.7
Glutamic Acid (% Total)	9.4	8.1	Valine (% Total)	3.5	2.8

Supplementary references

1. Guindon S, Dufayard J-F, Lefort V, Anisimova M, Hordijk W, Gascuel O. New algorithms and methods to estimate maximum-likelihood phylogenies: assessing the performance of PhyML 3.0. *Syst. Biol.* **59**, 307-321 (2010).
2. Larroude M, Celinska E, Back A, Thomas S, Nicaud JM, Ledesma‐Amaro R. A synthetic biology approach to transform *Yarrowia lipolytica* into a competitive biotechnological producer of β‐carotene. *Biotechnol. Bioeng.* **115**, 464-472 (2018).
3. Gao S*, et al.* Iterative integration of multiple-copy pathway genes in *Yarrowia lipolytica* for heterologous β-carotene production. *Metab. Eng.* **41**, 192-201 (2017).
4. Xie W, Ye L, Lv X, Xu H, Yu H. Sequential control of biosynthetic pathways for balanced utilization of metabolic intermediates in *Saccharomyces cerevisiae*. *Metab. Eng.* **28**, 8-18 (2015).
5. Yang J, Guo L. Biosynthesis of β-carotene in engineered *E. coli* using the MEP and MVA pathways. *Microb. Cell Fact.* **13**, 160 (2014).
6. Zhao J*, et al.* Engineering central metabolic modules of *Escherichia coli* for improving β-carotene production. *Metab. Eng.* **17**, 42-50 (2013).
7. Li Y*, et al.* Metabolic engineering of *Escherichia coli* using CRISPR–Cas9 meditated genome editing. *Metab. Eng.* **31**, 13-21 (2015).
8. Luo Z*, et al.* Enhancing isoprenoid synthesis in *Yarrowia lipolytica* by expressing the isopentenol utilization pathway and modulating intracellular hydrophobicity. *Metab. Eng.* **61**, 344-351 (2020).
9. Shi B*, et al.* Systematic metabolic engineering of *Saccharomyces cerevisiae* for lycopene overproduction. *J. Agric. Food. Chem.* **67**, 11148-11157 (2019).
10. Ma T*, et al.* Lipid engineering combined with systematic metabolic engineering of *Saccharomyces cerevisiae* for high-yield production of lycopene. *Metab. Eng.* **52**, 134-142 (2019).
11. Kang W*, et al.* Modular enzyme assembly for enhanced cascade biocatalysis and metabolic flux. *Nat. Commun.* **10**, 1-11 (2019).
12. Chen Y, Xiao W, Wang Y, Liu H, Li X, Yuan Y. Lycopene overproduction in *Saccharomyces cerevisiae* through combining pathway engineering with host engineering.

Microb. Cell Fact. **15**, 113 (2016).

13. Xie W, Lv X, Ye L, Zhou P, Yu H. Construction of lycopene-overproducing *Saccharomyces cerevisiae* by combining directed evolution and metabolic engineering. *Metab. Eng.* **30**, 69-78 (2015).
14. Sun T*, et al.* Production of lycopene by metabolically-engineered *Escherichia coli*. *Biotechnol. Lett.* **36**, 1515-1522 (2014).
