# Supplementary material for: Removal of lycopene substrate inhibition enables high carotenoid productivity in Yarrowia lipolytica
Source: Nat Commun. 2022 Jan 31;13:572. doi: 10.1038/s41467-022-28277-w (PMC8803881; doi:10.1038/s41467-022-28277-w)
Supplement: Supplementary file 4 — Reporting Summary [file 41467_2022_28277_MOESM4_ESM.pdf]

nature portfolio

Corresponding author(s): Gregory Stephanopoulos

Last updated by author(s): Jan 3, 2022

Reporting Summary

Nature Portfolio wishes to improve the reproducibility of the work that we publish. This form provides structure for consistency and transparency in reporting. For further information on Nature Portfolio policies, see our Editorial Policies and the Editorial Policy Checklist.

Statistics

For all statistical analyses, confirm that the following items are present in the figure legend, table legend, main text, or Methods section.

n/a	Confirmed	
☐	☒	The exact sample size (*n*) for each experimental group/condition, given as a discrete number and unit of measurement
☐	☒	A statement on whether measurements were taken from distinct samples or whether the same sample was measured repeatedly
☐	☒	The statistical test(s) used AND whether they are one- or two-sided<br>*Only common tests should be described solely by name; describe more complex techniques in the Methods section.*
☒	☐	A description of all covariates tested
☒	☐	A description of any assumptions or corrections, such as tests of normality and adjustment for multiple comparisons
☐	☒	A full description of the statistical parameters including central tendency (e.g. means) or other basic estimates (e.g. regression coefficient) AND variation (e.g. standard deviation) or associated estimates of uncertainty (e.g. confidence intervals)
☐	☒	For null hypothesis testing, the test statistic (e.g. *F*, *t*, *r*) with confidence intervals, effect sizes, degrees of freedom and *P* value noted<br>*Give P values as exact values whenever suitable.*
☒	☐	For Bayesian analysis, information on the choice of priors and Markov chain Monte Carlo settings
☒	☐	For hierarchical and complex designs, identification of the appropriate level for tests and full reporting of outcomes
☒	☐	Estimates of effect sizes (e.g. Cohen's *d*, Pearson's *r*), indicating how they were calculated

Our web collection on statistics for biologists contains articles on many of the points above.

Software and code

Policy information about availability of computer code

Data collection	LCsolution v1.25 SP4, Shimadzu for HPLC data quantification; Analyst v1.6.2 for LC-MS data acquisition; Agilent OpenLab Software 7890B for GC-FID data collection. Transform-restrained Rosetta (TrRosetta 3.9) server was used to create a computational structure model. PSI-blast 2.7.1 derives a position specific scoring matrix (PSSM) to generate evolutionary information. PAM30 was used to compute the substitution distance matrix. Agglomerative Clustering was used to cluster the variant sequence. ZEISS Axioskop v451487 with Nikon color camera was used to collect bright field microscopic images.
Data analysis	Prism9 v9.0.0 was used to plot data. TMHMM Server v.2.0 for prediction of transmembrane helices in protein; MAVEN v707 was used to process data collected from LC-MS measurements. PyMOL v1.8.6.2 was used for protein structure analysis. IsoCor v2.1.3 was used to correct the MS data for naturally occuring isotopes.

For manuscripts utilizing custom algorithms or software that are central to the research but not yet described in published literature, software must be made available to editors and reviewers. We strongly encourage code deposition in a community repository (e.g. GitHub). See the Nature Portfolio guidelines for submitting code & software for further information.

Data

Policy information about availability of data

All manuscripts must include a data availability statement. This statement should provide the following information, where applicable:

- Accession codes, unique identifiers, or web links for publicly available datasets
- A description of any restrictions on data availability
- For clinical datasets or third party data, please ensure that the statement adheres to our policy

All data supporting the findings of this study are available within the paper and its supplementary information files or from the corresponding author upon request.

The uniref90 database used in this study are available at https://www.uniprot.org/help/uniref. Source Data that support Figures 1b, 1c, 1d, 2b, 2c, 2e, 3b, 3c, 3d, 3e, 3f, 3g, 4b, 4c, 4d, 5a, 5b, 5c, 6a, 6d, S3b, S3c, S3d, S4, S8, S10, S12, S13, S14, S15, and S18 are provided. The gene sequence are available from NCBI with following accession number provided in the supplementary file: AY177424.1 (https://www.ncbi.nlm.nih.gov/nuccore/AY177424.1/); AJ238028.1 (https://www.ncbi.nlm.nih.gov/nuccore/AJ238028.1); AY177204.1 (https://www.ncbi.nlm.nih.gov/nuccore/AY177204.1); AJ250827.1 (https://www.ncbi.nlm.nih.gov/nuccore/AJ250827.1); AAA21260.1 (https://www.ncbi.nlm.nih.gov/protein/AAA21260.1); AF081514.1 (https://www.ncbi.nlm.nih.gov/nuccore/AF081514.1); D28748.1 (https://www.ncbi.nlm.nih.gov/nuccore/D28748.1); DQ016502.1 (https://www.ncbi.nlm.nih.gov/nuccore/DQ016502.1); XM_502923.1 (https://www.ncbi.nlm.nih.gov/nuccore/XM_502923.1/); D90087.2 (https://www.ncbi.nlm.nih.gov/nuccore/D90087.2); JX871358.1 (https://www.ncbi.nlm.nih.gov/nuccore/JX871358.1); CP002727.1 (https://www.ncbi.nlm.nih.gov/nuccore/CP002727.1); AY182008.1 (https://www.ncbi.nlm.nih.gov/nuccore/AY182008.1).

Field-specific reporting

Please select the one below that is the best fit for your research. If you are not sure, read the appropriate sections before making your selection.

- [x] Life sciences
- [ ] Behavioural & social sciences
- [ ] Ecological, evolutionary & environmental sciences

For a reference copy of the document with all sections, see nature.com/documents/nr-reporting-summary-flat.pdf

Life sciences study design

All studies must disclose on these points even when the disclosure is negative.

Sample size	As engineering was performed at the genetic level, and metabolite titers represent a large population of individual cells, sample size (n = 3) was determined based on the consistency of measurable differences between groups, where biological replicates represent independently well controlled microbial culture experiment.
Data exclusions	No data exclusion in this study
Replication	All determinations were biologically repeated at least twice. All attempts at replication were successful.
Randomization	For cell culture, single colonies of yeast strains were randomly selected to initiate the culture. All samples were measured randomly on the corresponding instruments.
Blinding	Blinding was not necessary since measurements did not involve bias from the experimentalist.

Reporting for specific materials, systems and methods

We require information from authors about some types of materials, experimental systems and methods used in many studies. Here, indicate whether each material, system or method listed is relevant to your study. If you are not sure if a list item applies to your research, read the appropriate section before selecting a response.

Materials & experimental systems

n/a	Involved in the study	
x		Antibodies
	x	Eukaryotic cell lines
x		Palaeontology and archaeology
x		Animals and other organisms
x		Human research participants
x		Clinical data
x		Dual use research of concern

Methods

n/a	Involved in the study	
x		ChIP-seq
x		Flow cytometry
x		MRI-based neuroimaging

Eukaryotic cell lines

Policy information about cell lines

Cell line source(s)	All Yarrowia lipolytica strains used in this study was based on the parental strain po1g that was purchased from Yeastern Biotech.
Authentication	Each Yarrowia lipolytica strain was confirmed by extracting genomic DNA and confirming the existence of key genes through PCR. Their proper morphology was also confirmed on agar plates and under the microscope.
Mycoplasma contamination	Yarrowia lipolytica strains were not tested for mycoplasma.
Commonly misidentified lines (See ICLAC register)	None
